# Machine Learning Quantified Tumor-Stroma Ratio Is an Independent Prognosticator in Muscle-Invasive Bladder Cancer

**DOI:** 10.3390/ijms24032746

**Published:** 2023-02-01

**Authors:** Qingyuan Zheng, Zhengyu Jiang, Xinmiao Ni, Song Yang, Panpan Jiao, Jiejun Wu, Lin Xiong, Jingping Yuan, Jingsong Wang, Jun Jian, Lei Wang, Rui Yang, Zhiyuan Chen, Xiuheng Liu

**Affiliations:** 1 Department of Urology, Renmin Hospital of Wuhan University, Wuhan 430060, China; 2 Institute of Urologic Disease, Renmin Hospital of Wuhan University, Wuhan 430060, China; 3 Department of Pathology, Renmin Hospital of Wuhan University, Wuhan 430060, China

**Keywords:** tumor-stroma ratio, whole slide image, machine learning, prognosis prediction, muscle-invasive bladder cancer

## Abstract

Although the tumor-stroma ratio (TSR) has prognostic value in many cancers, the traditional semi-quantitative visual assessment method has inter-observer variability, making it impossible for clinical practice. We aimed to develop a machine learning (ML) algorithm for accurately quantifying TSR in hematoxylin-and-eosin (H&E)-stained whole slide images (WSI) and further investigate its prognostic effect in patients with muscle-invasive bladder cancer (MIBC). We used an optimal cell classifier previously built based on QuPath open-source software and ML algorithm for quantitative calculation of TSR. We retrospectively analyzed data from two independent cohorts to verify the prognostic significance of ML-based TSR in MIBC patients. WSIs from 133 MIBC patients were used as the discovery set to identify the optimal association of TSR with patient survival outcomes. Furthermore, we performed validation in an independent external cohort consisting of 261 MIBC patients. We demonstrated a significant prognostic association of ML-based TSR with survival outcomes in MIBC patients (*p* < 0.001 for all comparisons), with higher TSR associated with better prognosis. Uni- and multivariate Cox regression analyses showed that TSR was independently associated with overall survival (*p* < 0.001 for all analyses) after adjusting for clinicopathological factors including age, gender, and pathologic stage. TSR was found to be a strong prognostic factor that was not redundant with the existing staging system in different subgroup analyses (*p* < 0.05 for all analyses). Finally, the expression of six genes (DACH1, DEEND2A, NOTCH4, DTWD1, TAF6L, and MARCHF5) were significantly associated with TSR, revealing possible potential biological relevance. In conclusion, we developed an ML algorithm based on WSIs of MIBC patients to accurately quantify TSR and demonstrated its prognostic validity for MIBC patients in two independent cohorts. This objective quantitative method allows application in clinical practice while reducing the workload of pathologists. Thus, it might be of significant aid in promoting precise pathology services in MIBC.

## 1. Introduction

Bladder cancer is one of the top 10 malignancies worldwide, with an estimated 573,000 new cases and 213,000 deaths in 2020 [1]. Once the tumor invades the muscle layer, the five-year survival rate for patients with muscle-invasive bladder cancer (MIBC) decreases dramatically. Even after radical cystectomy (RC), the five-year survival rate is only 40–60% [2,3,4]. Currently, the tumor-node-metastasis (TNM) staging established by the American Joint Committee on Cancer (AJCC) has high prognostic value and serves as the basis for clinical decision-making, but it is insufficient to cover the clinical characteristics of all MIBC patients [5]. Because MIBC is a heterogeneous disease, clinical outcomes can differ greatly among patients with the same TNM stage and treatment regimens [6]. Hence, it is necessary to find more reliable new prognostic biomarkers to guide personalized therapy for MIBC patients to avoid undertreatment or overtreatment. 

Tumor microenvironment (TME), also known as tumor-associated stroma, refers to the surrounding environment embedded in tumors that plays a crucial role in the occurrence, development, invasion, and metastasis of tumors, and is receiving increasing attention [7,8]. Emerging evidence suggests that tumor-stroma ratio (TSR) has a significant prognostic value in a variety of solid tumors, including MIBC, and high-abundant stroma is associated with poorer prognosis [9,10,11,12]. A study of MIBC transcriptome analysis revealed that higher stromal invasion was associated with shorter disease-specific survival even after neoadjuvant chemotherapy and RC [13]. The traditional semi-quantitative assessment of TSR is defined by pathologists under visual or microscopic examination, and a cut-off point of 50% is typically used as the basis for grouping [14]. Liu et al. assessed the TSR scores by visual inspection method and found that MIBC patients with a low stromal type (<50%) had a better prognosis [15]. However, traditional visual assessment methods are susceptible to the subjectivity of pathologists and inter-observer variability, preventing them from being widely used in clinical practice [16]. Furthermore, using a predefined cut-off point of 50% stromal content to classify patients into stromal-high or stromal-low groups to assess risk stratification of MIBC patients is not appropriate. Therefore, there is an urgent need to develop an automated method capable of achieving objective and standardized TSR quantification to facilitate accurate and efficient pathology services.

In recent years, advances in digital pathology and the development of artificial intelligence have led to the further quantitative analysis of thousands of available whole slide images (WSIs) [17]. Machine learning (ML) extracts high-order information from pathological images using manually predefined features to discover new biomarkers that are helpful for diagnosis and prognosis [18]. Pathological images-based ML analysis methods have proven to be useful for cancer detection [19], diagnosis [20], prognosis prediction [21], and molecular pattern recognition [22]. To further objectively evaluate the utility of TSR in tumor prognosis, ML-related automated quantification methods have been applied to breast cancer [23,24] and colorectal cancer [25,26]. However, the ML-based quantitative calculation method of TSR in MIBC has not been reported, which deserves further investigation.

In this study, we utilized a previously developed ML algorithm [27] and Qupath open-source software [28] to perform automated TSR assessment in histological slides for prognosis prediction in MIBC patients. We demonstrated in two independent cohorts that ML-based TSR is a robust independent prognostic factor that is not redundant with existing clinical and histopathological features. Finally, we further investigated the expression of the genes most correlated with TSR, revealing possible potential biological relevance.

## 2. Results

### 2.1. Patient Characteristics

Table 1 summarizes the baseline clinicopathological characteristics of the two cohorts. There were 56 deaths among 133 MIBC patients enrolled in the RHWU cohort and 124 deaths among 261 MIBC patients enrolled in the TCGA cohort. We selected only one representative WSI for each patient for analysis. We used the RHWU cohort as the discovery set and the TCGA cohort as an external validation set to evaluate the prognostic efficacy of TSR.

### 2.2. TSR Automated Assessment

**Figure 1:**
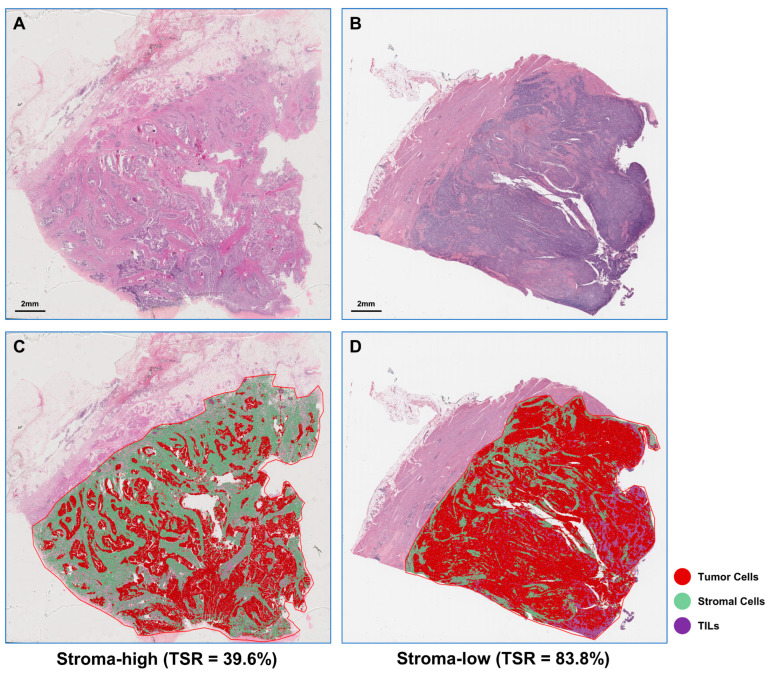
Representative examples of stromal-low (**A**) and stromal-high (**B**) H&E-stained WSI and corresponding machine learning algorithm segmentation results (**C**, stromal-low; **D**, stromal-high). H&E, hematoxylin-and-eosin; WSI, whole slide image; TILs, tumor-infiltrating lymphocytes.

We applied the trained cell classifier to WSIs to define tumor and stromal regions based on the distribution of four types of cells. Figure 1 shows representative examples when the ML algorithm is applied to the stroma-low and stroma-high WSI.

**Figure 2:**
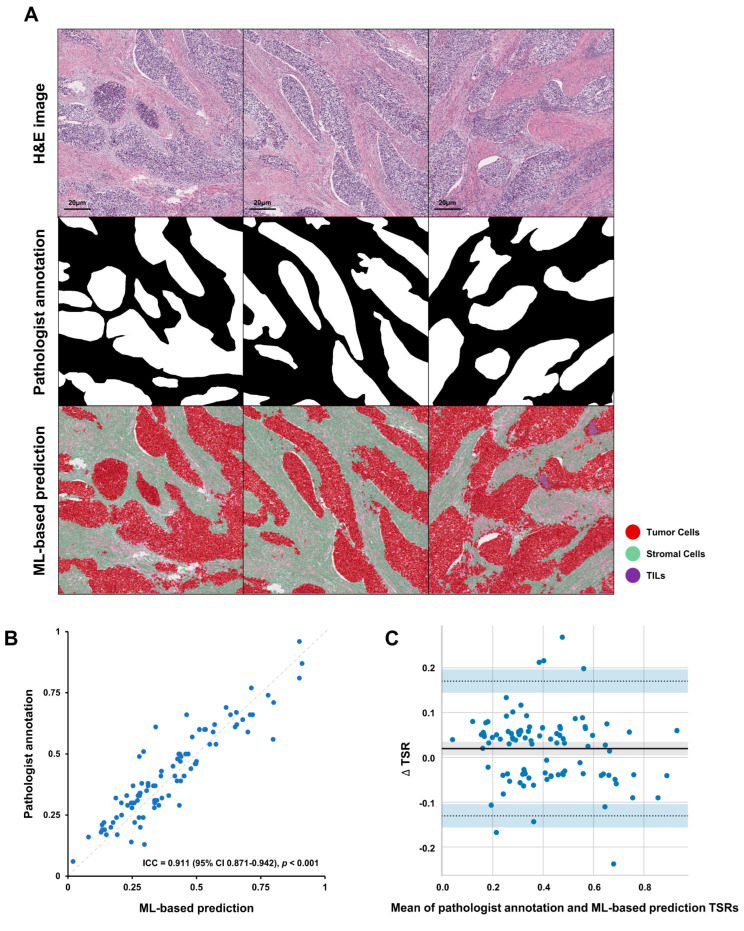
The results of TSR consistency analysis. (**A**) Examples of H&E images, pathologist annotations, and ML algorithm predictions. (**B**) Concordance between ML-based TSR and pathologist. (**C**) Bland-Altman plot for TSR estimation between ML algorithm and pathologist. The solid horizontal black line is the mean and the two dashed lines are ± SD. TSR, tumor-stromal ratio; HE, hematoxylin-and-eosin; ML, machine learning; TILs, tumor-infiltrating lymphocytes; SD, standard deviation.

For TSR consistency analysis, Figure 2A shows examples of manual annotation by pathologists and automatic segmentation and classification by ML algorithm. Good concordance was observed in the classification of tumor and stromal regions between the ML algorithm and pathologist annotation (Figure 2B). There was a high agreement between ML-based and annotated TSR (correlation = 0.911, 95% CI 0.871–0.942). Bland-Altman plot showed decent concordance between TSR calculated by the ML algorithm and that annotated by the pathologist, with a mean difference in TSRs of 0.02 (Figure 2C).

### 2.3. Evaluation of TSR as a Prognostic Variable in Two Cohorts

**Figure 3:**
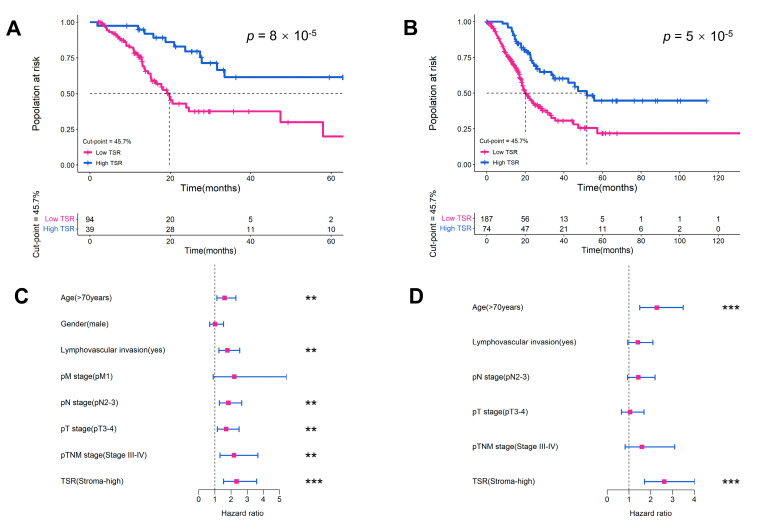
Evaluation of ML-based TSR with a 45.7% cut-off point as a prognostic variable in two cohorts. Kaplan–Meier survival curves for (**A**) RHWU and (**B**) TCGA cohort. Hazard ratio and 95% confidence interval for TSR and other clinicopathological features to predict survival in (**C**) univariate Cox and (**D**) multivariate Cox analyses. ML, machine learning. ***, *p* < 0.001; **, *p* < 0.01; *, *p* < 0.05.

We identified 45.7% as the optimal cut-off point for the RHWU cohort based on the X-tile software (Version 3.6.1) and divided patients into the low stroma (TSR ≥ 45.7%) or high stroma (TSR < 45.7%) groups. Stroma-high was identified in 94 (70%) and 187 (71%) patients in the RHWU cohort and TCGA cohort, respectively. Our results showed that low levels of TSR were associated with shorter overall survival (OS) in the RHWU cohort (hazard ratio [HR] = 2.851, *p* < 0.0001; Figure 3A) and the TCGA cohort (HR = 2.346, *p* < 0.0001; Figure 3B). We performed uni- and multivariate Cox analyses in the TCGA cohort to assess associations of TSR and clinicopathological features with prognosis. In univariate Cox analysis, TSR (with a predefined 45.7% cut-off point), age, lymphovascular invasion, pT stage, pN stage, and pTNM stage were all significantly associated with OS (Figure 3C). Multivariate Cox analysis showed that TSR remained a significant prognostic factor after retaining significant prognostic indicators in univariate analysis (HR = 2.622, log-rank *p* < 0.001; Figure 3D and Table 2).

### 2.4. Validation of the Prognostic Value of TSR in Different Subgroups

**Figure 4:**
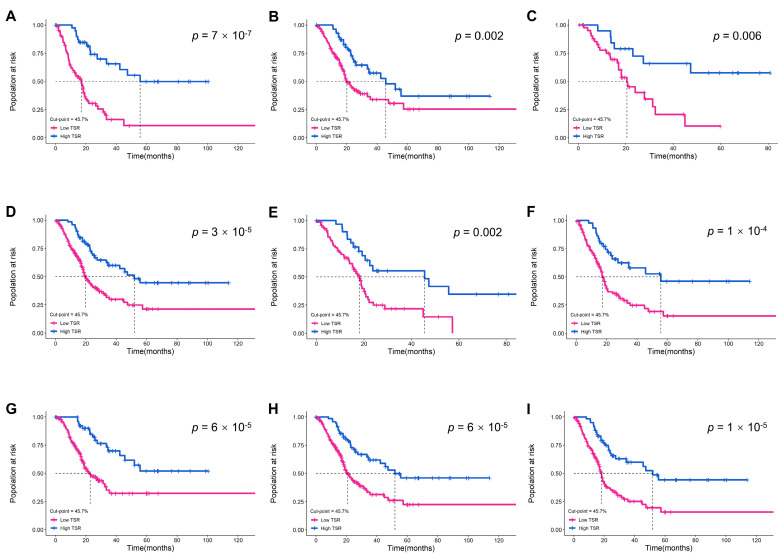
The performance of ML-based TSR with a 45.7% cut-off point in predicting prognosis in the TCGA cohort. Kaplan–Meier survival curves for the following subgroups: (**A**) age ≥ 70; (**B**) male; (**C**) female; (**D**) high histologic grade; (**E**) lymphovascular invasion; (**F**) pT stage 3–4; (**G**) pN stage 0–1; (**H**) pM stage 0; (**I**) pTNM stage III-IV.

We analyzed MIBC patients in the TCGA cohort to further investigate the prognostic value of TSR with a cut-off point of 45.7% in different subgroups. The results confirmed that higher TSR was not only associated with better prognosis in pTNM stage III-IV patients, but also enabled risk identification in subgroups with other characteristics (age, gender, pT stage, pN stage, pM stage, pTNM stage, and lymphovascular invasion) (Figure 4). Therefore, ML-based TSR is not redundant with the existing staging system, and could further implement risk stratification for high-risk stage III-IV MIBC patients, and facilitate more aggressive treatment decisions.

### 2.5. Gene Expression Correlation with TSR

**Figure 5:**
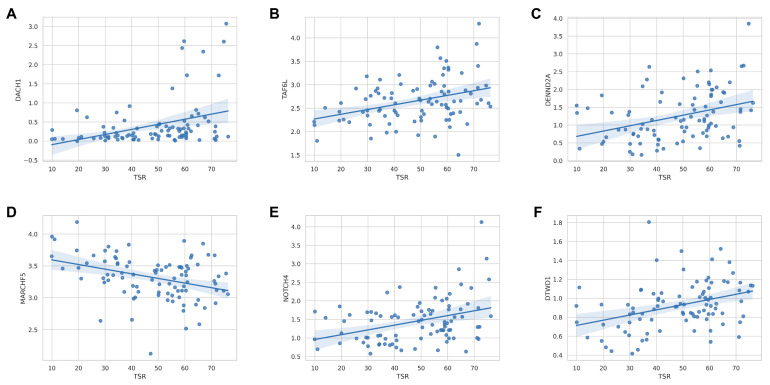
Correlates between ML-based TSR and gene expression levels. Biological correlation between TSR and (**A**) the DACH1 expression (N = 92 samples), (**B**) the TAF6L expression (N = 91 samples), (**C**) the DENND2A expression (N = 92 samples), (**D**) the MARCHF5 expression (N = 92 samples), (**E**) the NOTCH4 expression (N = 92 samples), and (**F**) the DTWD1 expression (N = 92 samples) available for the TCGA dataset.

The TCGA database contains molecular and gene expression information of MIBC patients, allowing us to investigate the correlation between TSR and genomics. After excluding missing values, we included 92 MIBC patients. Associations between TSR and gene expression levels in each patient were examined by Spearman correlation analysis, revealing possible potential biological correlations. The results showed that the expression of six genes was significantly correlated with TSR: DACH1 (correlation = 0.401; Spearman’s correlation test, *p* = 4.2 × 10^−4^), TAF6L (correlation = 0.361; Spearman’s correlation test, *p* = 2.6 × 10^−3^), DENND2A (correlation = 0.414; Spearman’s correlation test, *p* = 2.3 × 10^−4^), MARCHF5 (correlation = −0.370; Spearman’s correlation test, *p* = 1.3 × 10^−3^), NOTCH4 (correlation = 0.376; Spearman’s correlation test, *p* = 1.5 × 10^−3^) and DTWD1 (correlation = 0.438; Spearman’s correlation test, *p* = 8.4 × 10^−5^) (Figure 5).

## 3. Discussion

Bladder cancer can be divided into non-muscle invasive bladder cancer and MIBC according to the TNM staging system [29]. Currently, cisplatin-based neoadjuvant chemotherapy is the standard treatment for pre-RC MIBC [30]. Despite the fact that aggressive local therapy has the potential to eliminate residual cancer and improve survival in patients with locally advanced bladder cancer, clinical benefit is unlikely in approximately 60% of patients, with potentially fatal surgical delays and treatment toxicity [31]. The TNM staging system is typically used to predict the prognosis of MIBC patients, but there are also some poorly differentiated tumors that have a very poor prognosis regardless of the TNM stage [32]. Thus, there is an urgent need to develop more new prognostic prediction methods independent of the current TNM staging for MIBC patients. In this study, we performed accurate TSR quantification of WSIs in MIBC patients using a previously proposed ML algorithm and validated its prognostic value independent of the TNM system.

The TME is composed of a variety of immune cells, stromal cells, and the factors they secrete, cultivating an intratumoral atmosphere of chronic inflammation, immunosuppression, and pro-angiogenic [33]. An increasing number of studies have demonstrated that TSR in the TME has a crucial prognostic effect on various solid tumors [15,34,35,36]. Unfortunately, traditional visual or microscopic methods are susceptible to inter-institutional or inter-observer variability, making them unavailable in clinical practice. Recently, some studies have attempted to quantify TSR using ML or convolutional neural network methods, thereby revealing the correlation with the prognosis of cancer patients [24,25,26,37]. However, such methods have not been validated in MIBC patients and cannot provide an optimal cut-off point for TSR, which is critical for clinical applicability.

In the present work, we used an ML approach to quantitatively calculate TSR and validated that high stromal tumors (low TSR) were associated with poorer prognosis in two independent cohorts of MIBC patients. To the best of our knowledge, our study provides the first evidence of the prognostic effect of quantitative TSR in MIBC, which will aid in personalized therapy. Our results in subgroups demonstrated that TSR is not redundant with existing clinical, biological, and histopathological features and is a strong prognostic factor. This method allows us to perform objective and reliable TSR assessments of WSIs while reducing the workload of pathologists, making it suitable for use in clinical practice. Furthermore, compared with traditional visual assessment methods, ML-based TSR is a continuous variable rather than a discrete classification, which could guide us to use a more appropriate predefined cut-off point for risk stratification. But in this study, TSR was not adjusted for specific pathological stages, tumor molecular subtype, variant histology, and treatment modality. Therefore, we suggest that the cut-off point of TSR can be defined with an appropriate range, so that it can be adjusted according to the actual situation in future large-scale clinical trials and practice.

With the development of next-generation sequencing technology and bioinformatics, more and more effective biomarkers have been discovered [38]. Programmed cell death ligand 1-based immunohistochemical detection is probably the most widely accepted method [39]. However, the implementation of high-throughput gene sequencing technology as well as immunohistochemical staining methods in clinical practice has been hampered by high costs, tissue preparation requirements, and issues of standardization and reproducibility [40]. In contrast, our ML method only requires TSR quantification on a single WSI to perform prognostic analysis on MIBC patients. Because such histological materials are readily available in the surgical setting, the ML method is easier to carry out in clinical practice management. Moreover, an ML-based TSR quantification method that is unaffected by inter-institutional and inter-observer variability will hopefully result in a reliable, reproducible, and standardized prognostic strategy.

We also further explored the association between TSR and gene expression levels in MIBC patients, and found that DACH1, DEEND2A, NOTCH4, DTWD1, and TAF6L were positively correlated with TSR, while MARCHF5 was negatively correlated. Some of these six genes have been reported to play important roles in regulating a range of cellular processes, including tumor occurrence and progression, as previously reported. DACH1, a known tumor suppressor gene in breast, colon, and kidney cancers, plays a key role in tumor growth and metastasis by acting on cell cycle control [41,42,43]. NOTCH4, one of four transmembrane receptors in the NOTCH family, is frequently mutated in several cancer types. A recent study reported that NOTCH4 mutation is a novel biomarker associated with better response to immune checkpoint inhibitor therapy in a variety of cancers, including bladder cancer [44]. TAF6L is identified as a novel epigenetic regulator of the embryonic stem cell state. Together with c-MYC, TAF5L/TAF6L activates the MYC regulatory network, which primarily regulates the cell cycle, DNA replication, ribosome biosynthesis, and metabolism, as well as maintaining embryonic stem cell proliferation and self-renewal [45]. The tumor suppressor gene DTWD1, a novel p53 target gene, inhibits cancer cell growth by reducing the expression of cyclin B1 [46,47]. MARCH5 belongs to the MARCH family and is an integral mitochondrial outer membrane protein involved in the control of mitochondrial morphology [48]. A study has reported that MARCH5 is more highly expressed in epithelial ovarian cancer tissues and promotes tumor migration, invasion, and autophagy [49]. The above findings show that through the analysis of TSR combined with genomics, some genes closely related to tumor occurrence and progression can be identified, providing a reference for the discovery of more new biomarkers and therapeutic targets.

There are still limitations to our work. First, our study was retrospective, which has inherent shortcomings. The endpoint of both cohorts was overall survival, and the prognostic value of disease-free survival was not analyzed. Thus, it is necessary to carry out a multi-center prospective clinical trial to further explore the prognostic efficacy of ML-based TSR and human-determined TSR, and the association of this variation with survival. Second, while we used the ESV function in our study to unify the staining differences of different WSIs, the impact of such differences on cell segmentation or classification could not be completely eliminated. To improve the quality of pathological slide images in the future, a standardized production procedure from sample collection to digital scanning will be required. Finally, manual annotations about tumor regions are not automatic; Instead, annotations are performed subjectively by the pathologist. As such, different pathologists will receive different TSR scores, because their annotations will not be exactly the same. To address this issue in the future, we will require a detection module that can objectively and automatically annotate tumor regions.

## 4. Materials and Methods

### 4.1. Patient Cohorts

In this study, we retrospectively analyzed two independent cohorts. The first cohort was from The Cancer Genome Atlas (TCGA) and included 457 WSIs from 386 patients with bladder cancer (https://portal.gdc.cancer.gov/, accessed on 2 December 2022). The second cohort, from Renmin Hospital of Wuhan University (RHWU; Wuhan, Hubei, China), included 150 WSIs from 150 patients with bladder cancer diagnosed from 2017 to 2022. All pathological slide images were saved in the form of digital WSI.

Inclusion criteria for both cohorts were as follows: (a) specific pathological diagnosis of MIBC, (b) available clinicopathological information, (c) available follow-up information, and (d) availability of clear H&E-stained diagnostic slides.

In addition, we also collected the clinical data, biological and pathological characteristics (including age, gender, lymphovascular invasion, survival status, survival time, pathological grade, and TNM stage (according to the 8th edition of the AJCC staging manual [50])) of patients in the two cohorts. Among them, the patient data of the TCGA cohort can be obtained through the UCSC Xena database (http://xena.ucsc.edu/, accessed on 2 December 2022), and the patient data of the RHWU cohort can be obtained through the hospital information management system.

### 4.2. Ethics

This retrospective study was approved by the RHWU Ethics Committee (No. WDRY2022-K084), and informed consent was obtained from the patients.

### 4.3. WSI Preprocessing

H&E-stained slides from the RHWU cohort were digitized using a KF-PRO-020 digital scanner at 20× magnification (0.5 µm per pixel). After the scanning was completed, all WSIs were carefully reviewed by a uropathologist to ensure that all images were clear and usable prior to further analysis, followed by annotation of tumor regions. Since the WSIs of the two cohorts were at different magnifications, we uniformly loaded them at 20× magnification here. All WSIs were annotated with tumor regions by one pathologist and reviewed by another pathologist before further analysis.

### 4.4. WSI Image Analysis

**Figure 6:**
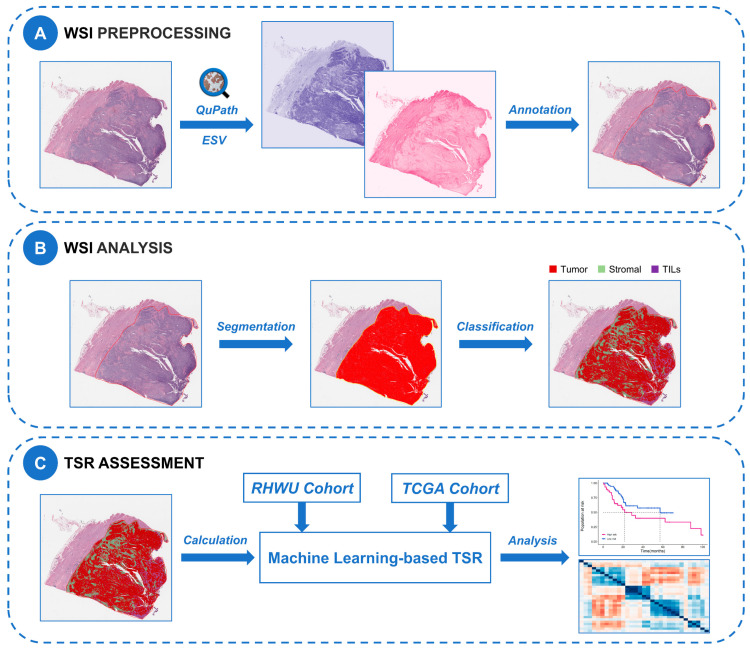
Flow chart of ML-based WSI processing and analysis in this study. (**A**) WSI reprocessing. All WSIs were loaded in QuPath, normalized for staining with the ESV function, and annotated by pathologists. (**B**) WSI analysis. We used the watershed cell detection method for cell segmentation, and then trained an optimal neural network for cell classification. (**C**) TSR assessment. The number of cells in all categories was counted to calculate TSR, and the correlation of TSR with prognosis and gene expression association was analyzed in two dependent cohorts. WSI, whole slide image; ESV, estimated staining vector; TSR, tumor-stroma ratio; TILs, tumor-infiltrating lymphocytes.

In this study, we used the ML algorithm based on the QuPath open-source software (Version 0.3.2) with the neural network method and the optimal cell classifier in the previous study [27] for cell segmentation and classification. Due to staining variability in histological slides both between cohorts and within cohorts, we optimized H&E staining intensity for each WSI using the estimated staining vector (ESV) function in QuPath. The workflow for staining normalization using the ESV function is shown in Supplementary Figure S1. We used the watershed cell detection method to perform cell segmentation, and the parameters involved were set as follows: Detection image: hematoxylin OD; requested pixel size: 0.5 µm; background radius: 8 µm; median filter radius: 0 µm; sigma: 1.5 µm; minimum cell area: 10 µm^2^; maximum cell area: 400 µm^2^; threshold: 0.1; maximum background intensity: 2. For cell classification, an expert pathologist selected representative specific regions to classify tumor cells (red), TILs (purple), and stromal cells (green), with the remaining irrelevant factors (false detections and background) set to “ignore”. Then based on these representative regions, we used the built-in neural network classifier with 8 hidden layers (maximum iterations: 1000) for training to produce the optimal cell classification. To further improve classification accuracy, we added specific regions as needed and smooth object features at 25 µm and 50 µm radius to complement the existing measurement features of cells. Supplementary Table S1 described all features used for cell classification. The training of the classifier required multiple rounds of optimization to achieve the best classification effect. All the above processes were quality controlled by uropathologists. We applied the optimal cell classifier to all WSIs in both cohorts via a built-in script in QuPath which could be available in an online repository (https://github.com/zqy396/ML_TSR/, accessed on 22 January 2023) to improve reproducibility, and then counted the number of each cell type. Finally, the flowchart of this study is shown in Figure 6.

### 4.5. TSR Assessment

In this study, the TSR defined by ML was calculated as: TSR = tumor cells/(stroma cells + tumor cells) × 100%. We calculated TSR based on the counted four types of cells as quantitative results.

To assess the concordance of TSR estimation between the ML algorithm and pathologist annotation, we used random 100 WSIs from the TCGA cohort for consistency analysis, and then calculated the Spearman correlation coefficient and intra-class correlation coefficient (ICC). The Bland-Altman plot was used to determine the agreement of TSR estimation between the ML algorithm and pathologist annotation.

### 4.6. Statistical Analysis

We used SPSS 26.0 software (SPSS Inc., Chicago, IL, USA) for statistical analysis. The X-tile cut-point finder [51], a software that traverses possible combined partitions to find the optimal classification threshold, was used to determine statistical significance thresholds for TSR. Kaplan-Meier survival curves were drawn using R software (Version 3.5.1) for prognostic analysis, and log-rank tests were performed. The prognostic value of TSR was evaluated using uni- and multivariate Cox proportional hazards models. A Cox model with TSR, age, gender, lymphovascular invasion, and TNM stage as variables was generated for univariate analysis. All *p* values were two-tailed, and *p* values less than 0.05 were considered statistically significant.

## 5. Conclusions

We presented an ML method for the quantitative assessment of TSR using digitized H&E-stained images of MIBC. We demonstrated in two independent cohorts that ML-based TSR is a robust prognostic factor that is not redundant with existing prognostic factors. Quantitative and standardized analysis of histopathological images by ML algorithms might be of great help in clinical prognosis prediction and decision-making of MIBC patients.

## Figures and Tables

**Table 1:** The distributions of demographic and clinicopathologic characteristics of MIBC patients in the two cohorts.

	RHWU (N = 133)	TCGA (N = 261)
Age (years)	66 (26, 87)	69 (37, 90)
Sex		
female	20 (15.04%)	64 (24.52%)
male	113 (84.96%)	197 (75.48%)
pT stage		
pT2	52 (39.10%)	75 (28.74%)
pT3	63 (47.37%)	141 (54.02%)
pT4	18 (13.53%)	40 (15.33%)
pTx	0 (0%)	5 (1.92%)
pN stage		
pN0	66 (49.62%)	151 (57.85%)
pN1	34 (25.56%)	34 (13.03%)
pN2	18 (13.53%)	58 (22.22%)
pN3	15 (11.28%)	5 (1.92%)
pNx	0 (0%)	13 (4.98%)
pM stage		
pM0	129 (96.99%)	112 (42.91%)
pM1	4 (3.01%)	7 (2.68%)
pMx	0 (0%)	142 (54.41%)
pTNM stage		
Stage II	38 (28.57%)	65 (24.90%)
Stage III	74 (55.64%)	95 (36.40%)
Stage IV	21 (15.79%)	101 (38.70%)
Lymphovascular invasion		
No	83 (62.41%)	80 (30.65%)
Yes	50 (37.59%)	102 (39.08%)
Missing	0 (0%)	79 (30.27%)
Survival status		
Alive	77 (57.89%)	137 (52.49%)
Dead	56 (42.11%)	124 (47.51%)
OS time (months)	15.3 (1.9, 66.0)	17.4 (0, 132.7)

MIBC, Muscle-invasive Bladder Cancer; OS, Overall survival.

**Table 2:** Uni- and multivariate Cox analyses of prognostic factors in the TCGA cohort.

	Univariate Analysis		Multivariate Analysis	
	HR (95% CI)	*p*-Value	HR (95% CI)	*p*-Value
Age				
<70	Ref.		Ref.	
≥70	1.610 (1.127, 2.298)	0.009	2.281 (1.493, 3.487)	<0.001
Gender				
female	Ref.			
male	1.020 (0.679, 1.533)	0.923		
pT stage				
pT1-2	Ref.		Ref.	
pT3-4	1.700 (1.157, 2.499)	0.007	1.052 (0.654, 1.692)	0.552
pN stage				
pN0-1	Ref.		Ref.	
pN2-3	1.842 (1.275, 2.662)	0.001	1.433 (0.934, 2.197)	0.142
pM stage				
pM0	Ref.			
pM1	2.211 (0.899, 5.439)	0.084		
pTNM stage				
Stage II	Ref.		Ref.	
Stage III-IV	2.193 (1.314, 3.660)	0.003	1.598 (0.826, 3.094)	0.138
Lymphovascular invasion				
No	Ref.		Ref.	
Yes	1.779 (1.249, 2.533)	0.001	1.410 (0.947, 2.100)	0.070
TSR				
Stroma-low	Ref.		Ref.	
Stroma-high	2.346 (1.537, 3.581)	<0.001	2.622 (1.712, 4.015)	<0.001

95% CI, 95% Confidence Interval.

## Data Availability

The datasets of TCGA cohort for this study can be found in the [The Cancer Genome Atlas Program] [https://portal.gdc.cancer.gov/, accessed on 10 December 2022]. All scripts involved in the study can be accessed in the online repository [ML_TSR] [https://github.com/zqy396/ML_TSR/, accessed on 22 January 2023].

## Supplementary Materials

The following supporting information can be downloaded at: https://www.mdpi.com/article/10.3390/ijms24032746/s1. Supplementary Figure S1. The workflow for staining normalization using the estimated staining vector function in QuPath. (A) A representative region is selected for staining vector estimation for the entire image. (B) Estimate stain vector using the “Auto” function. (C) Eosin stain vector before staining normalization and (D) after normalization. (E) Hematoxylin stain vector before staining normalization and (F) after normalization. Supplementary Table S1. Features incorporated by neural network-driven cell classifier.
